# Sanctions or National Policies in COVID-19 Management in Iran: Which One is More Effective?!

**DOI:** 10.30476/beat.2021.90365.1249

**Published:** 2021-10

**Authors:** Mahmoudreza Peyravi, Milad Ahmadi Marzaleh

**Affiliations:** 1 Department of Health in Disasters and Emergencies, School of Management and Medical Informatics, Shiraz University of Medical Sciences, Shiraz, Iran

**Dear Editor**

The new corona disease (COVID-19) is very contagious and features a high risk of vast outbreak and infection. The World Health Organization (WHO) declared a global public health emergency condition on 30 January 2020 and a universal epidemic afterwards by given its rapid epidemic worldwide (1). Iran was amongst the first countries influenced by COVID-19. Iran’s health system was shocked by the fast and vast outbreak of the virus which made it impossible to take appropriate measures on the first days of COVID-19 outbreak for its management. However, Iran made maximal efforts returning to normal conditions. On the other hand, United States (US) sanctions contributed to the worsening of the conditions. The sanctions that mark the US’s economic and political war against Iran, date back to long time ago. These sanctions had indirect, adverse effects on the relief and rescue measures during the 2019 flood in Iran, as well (2). Sanctions are the perfect violation of humanitarian programs and are in conflict with the United Nations (UN) charter and the international human right declaration. Nonetheless, not only COVID-19 had no effects on the US’s perspectives and policies, but the sanctions have also been increased by the US in this period. In fact, the sanctions are the genuine manifestation of the US’s application of terrorism. These sanctions exert subtle physical, psychological, and mental effects on the people based on contribution to further spread of COVID-19.

Most problems in Iran appeared on the first days of COVID-19 outbreak. In the beginning of the COVID-19 outbreak, Iran was needed medical and sanitary aids. However, the country was self-efficient to produce personal protection in the two months after diagnosis of the COVID-19 first case includes shield, gloves, mask, protective gear, and antiseptic materials. Hospitals, private and public treatment centers took effective measures by increasing the number of their ICU beds and equipping them with the required equipment like ventilators in the regions with high outbreak of the disease. Extra beds were also made by the Red Crescent Society and military forces as well as the other related institutions for the patients’ convalescence period. Creation of several field hospitals was also amongst the very appropriate interventions during the response phase. At present, Iran’s efforts are admirable to do extensive research on producing COVID-19 vaccine.

Getting over COVID-19 entails global consensus and participation of all countries as it was first appeared in Wuhan, China and spread in the whole world. Thus, national efforts and solidarity to cover COVID-19 at the international level are of great importance. However, sanctions have indirect, but not strong and serious effects on responding to and managing COVID-19. Nonetheless, national and international policies influence all aspects of people’s health in short and long run. Local governments are primarily responsible for safeguarding their people’s health, and violation of the national duty is not justifiable by the sanctions negative effects. Reinforcement of the economic, cultural, political, and healthcare infrastructures helps overcome the health shocks and newly emerging diseases like COVID-19. Through strengthening the people’s health, the countries’ empowerment can be augmented and they can be encouraged to become self-efficient and concentrate on their competencies by community-oriented interventions and evidence-based decision-making, thereby, safeguarding their national securities.

The following solutions can be suggested to overcome and reduce the effects of the sanctions and corroborate the infrastructures and national policies: 1) empowerment of economic, cultural, and political infrastructures as well as national and local policies, 2) entry of non-governmental and impartial organizations like Red Cross and Red Crescent into the exchange of healthcare and treatment equipment to the countries inflicted with sanctions, 3) decisive entry by the UN into the arena of the dismissal of all sanctions, 4) convergence of the neighboring and regional countries to respond the COVID-19 and overcome the sanctions, 5) concentration on healthcare and empowerment of domestic economy, 6) corroboration of the foreign policy to get prepared for post-COVID-19, 7) complete enforcement of the international agreements and conventions, including Joint Comprehensive Plan of Action (JCOPA), 8) arrangement of moderated political relations between Iran and the US by the UN, 9) the experiences transfer on the fight against sanctions by the allied countries, 10) the third intermediation and impartial countries to remove sanctions such as Switzerland, 11) the sanctions threats transformation to proper opportunities by politicians, 12) change in the power of pharmaceutical and healthcare-related productions by European and American countries, China, and India, 13) exercising realism on display of the sanctions effects and concentration on the local and national interventions in Iran, and 14) institutionalization of the national beliefs in the countries’ self-reliance.

National and international collaborations for preventing and controlling COVID-19 are very important by considering the daily increasing spread of the COVID-19 pandemic around the globe. Nevertheless, sanctions have exerted a considerable effect on COVID-19 control through weakening the economic resources and creating disruption in the process of drug and equipment transfer. However, national efforts play more considerable and vital role in COVID-19 management. Hence, healthcare policymakers and decision-makers in Iran and other countries afflicted with sanctions should elevate their national empowerment, make firm decisions, corroborate and concentrate on research and development as well as health-oriented and pharmaceutical industries, engage in acculturalization to take steps towards controlling and managing COVID-19, and become the new global players with the maximum influence in the area of COVID-19 management.

## References

[1] Huang C, Wang Y, Li X, Ren L, Zhao J, Hu Y (2020). Clinical features of patients infected with 2019 novel coronavirus in Wuhan, China. Lancet.

[2] Peyravi M, Ahmadi Marzaleh M (2020). The Effect of the US Sanctions on Humanitarian Aids during the Great Flood of Iran in 2019. Prehosp Disaster Med.

